# Effective Connectivity Reveals Strategy Differences in an Expert Calculator

**DOI:** 10.1371/journal.pone.0073746

**Published:** 2013-09-23

**Authors:** Ludovico Minati, Natasha Sigala

**Affiliations:** 1 Brighton and Sussex Medical School, University of Sussex, East Sussex, United Kingdom; 2 Scientific Department, Fondazione IRCCS Istituto Neurologico “Carlo Besta”, Milano, Italy; 3 Sackler Centre for Consciousness Science, University of Sussex, East Sussex, United Kingdom; Duke University, United States of America

## Abstract

Mathematical reasoning is a core component of cognition and the study of experts defines the upper limits of human cognitive abilities, which is why we are fascinated by peak performers, such as chess masters and mental calculators. Here, we investigated the neural bases of calendrical skills, i.e. the ability to rapidly identify the weekday of a particular date, in a gifted mental calculator who does not fall in the autistic spectrum, using functional MRI. Graph-based mapping of effective connectivity, but not univariate analysis, revealed distinct anatomical location of “cortical hubs” supporting the processing of well-practiced close dates and less-practiced remote dates: the former engaged predominantly occipital and medial temporal areas, whereas the latter were associated mainly with prefrontal, orbitofrontal and anterior cingulate connectivity. These results point to the effect of extensive practice on the development of expertise and long term working memory, and demonstrate the role of frontal networks in supporting performance on less practiced calculations, which incur additional processing demands. Through the example of calendrical skills, our results demonstrate that the ability to perform complex calculations is initially supported by extensive attentional and strategic resources, which, as expertise develops, are gradually replaced by access to long term working memory for familiar material.

## Introduction

The study of calendrical skills, i.e. the ability to rapidly name the weekday corresponding to a particular date, is usually confined to savants, who are individuals gifted with specific skills but often affected by deficits in social and other intellectual domains [1]. The ability to mentally perform calendrical calculation seems to be an acquired trait, developed as a consequence of obsessive preoccupation with calendars through extraction of rules and regularities, e.g. 28-year and 400-year cyclical repetitions [2], [3], [4]. Biological factors may predispose to and facilitate the acquisition of such skills; for example, abnormal connectivity in autism leads to over-representation of details and dampened semantic integration, potentially biasing attention to specific problems from which one would otherwise be easily distracted by other stimuli (Snyder 2009, Wass 2011). However, calendrical skills are occasionally found even in cognitively and behaviourally normal individuals: while this is a rare occurrence, plausibly because of lack of interest and motivation, it demonstrates that such skills can be acquired [5]; notable examples include Professor A. C. Aitken [6], [7] and Professor Conway [3], both distinguished academic mathematicians.

Mental calculation experts have been previously studied in the context of skilled memory theory, which posits that their abilities are underpinned by increased working memory capacity for materials within an area of expertise, and result from sustained practice and learning to encode and retrieve relevant information in long term memory (LTM). In the case of experts it has been proposed that part of LTM is used in working memory tasks, which overcomes the limitations of working memory and speeds up performance [8], giving rise to long-term working memory [9]. The suggestion that the remarkable skills of mental calculators result from their superior capacity to hold information in long-term working memory and a vast repertoire of computational strategies, would imply that most individuals are, in principle, able to adopt specific strategies to extend their limits of performance in certain reasoning domains. Empirical support for the view that experts are made, not born, comes for instance from Staszewski's experiments, in which two undergraduate students were trained in mental calculations over a three-year period (with at least 175 hours of practice) [10]. Such training resulted in a dramatic reduction of solution times, which was attained in part through improved memory performance and in part through discovery and adoption of efficient strategies for solving increasingly complex two-place multiplication problems.

Previous imaging studies of calendrical savants and calculator prodigies have attempted to dissociate whether their skills rely on memory or calculation. In an early PET study [11], arithmetic but not calendrical calculations were associated with increased activation of right prefrontal and medial temporal areas in a calculator prodigy (CP, real name R. Gamm) compared to six control participants. This finding supported the theory of long-term working memory [9], which posits that storage of information in one's area of expertise relies on the association of encoded information and retrieval of LTM cues, by demonstrating a selective involvement of brain areas associated with episodic memory in CP only. These activations are thought to allow easy storage and retrieval of intermediate results from memory during calculations. In line with this view, the first PET study of an autistic calendrical savant also found activations in frontal and temporal areas, consistent with memory processing [12].

By contrast, the first functional MRI (fMRI) study of two calendrical savants [5], reported increased bilateral activation in superior (BA 7) and inferior (BA 40) parietal areas during calendric compared to arithmetic calculation. Differential responses to the tasks were additionally found in premotor and supplementary motor regions for one participant, and in inferior temporal cortex in the other. Furthermore, a parametric increase in activation of the parietal areas with respect to task difficulty, manipulated by increasing date distance from the present and indexed by reaction times [13],[14], was demonstrated. A more recent fMRI study directly compared a self-taught mathematical prodigy, CP, also scanned by [11], with an autistic savant: activation clusters in the former were principally found in frontal and parietal areas, and co-localized with those reported by [5], however in the latter the activation pattern was scattered and included occipital, parietal, frontal, thalamic and cerebellar areas [15]. Although this result should be interpreted with some caution, as the autistic participant had difficulties with the experimental setup, there appears to be substantial inter-individual variation in the anatomical substrates of calculation as performed by experts in the same domain, which could result from their different ages, learning histories or strategies applied to solve the same problems.

Given the relative rarity of these cases, the exact determinants of the observed brain activity differences remain unclear, but it is reasonable to assume that different strategies were adopted to solve the same calculations [16], [17], [18]; indeed, broader behavioural investigations of savants have demonstrated that their specific skills are not acquired in a consistent manner or associated with particular cognitive constructs, but rather emerge in an unpredictable manner throughout development, e.g. [19], [20].

In the present study, we explicitly investigate whether even a single individual with specific calculation skills may adopt different strategies to solve problems characterized by different features. We studied a non-autistic, exceptional calendar calculator (YV), who can correctly identify up to 93 weekdays for past and future dates in one minute. We hypothesised that his strategy would change as a function of calculation difficulty and/or familiarity, and that this would reflect in differentiable anatomical localization of activity as well as in whole-brain connectivity. Specifically, we expected stronger univariate activations of middle temporal lobe structures (hippocampal and parahippocampal areas), associated with episodic encoding and retrieval, as well as information maintenance across delays [11], (Young, Otto et al. 1997), (Olsen, Nichols et al. 2009), during easier/more practiced calculations. To this end, we applied traditional univariate analysis and graph-based network mapping. The latter can reveal the involvement of regions that appear silent or near-silent on univariate correlation maps, cf. [21], [22], and supports the identification of “cortical hubs” from functional MRI data. Combination of these two approaches allowed us to model brain activity not only in terms of individual activations but also of network architecture and neural context [23], [24], overcoming the limitations inherent in the localization approach adopted in previous work in this area.

## Methods

### Participant

The study participant, YV, is a left-handed, 30-year-old native Spanish speaker with postgraduate-level education in mathematics and computer science. He is an extremely skilled calendar calculator, and can supply up to 93 correct weekdays to given dates in 60 seconds. YV rated the items on the Autism spectrum Quotient (AQ; [25]) and his score of 13 is well below the threshold of 32 associated with high functioning autism or Asperger's syndrome. He also completed a computerized version of the Raven's Progressive Matrices Test [26], as a validated, non-verbal measure of reasoning ability that consists of the visual completion of geometric designs, e.g. [27], and which has been shown to correlate with general intelligence [28]. YV scored 157 at this test, which is 15 standard deviations higher than the average score of 98.4. Unlike many previously studied savants and the expert CP, who are characterized by social interaction difficulties and emotional idiosyncrasies [15], [29], YV appeared extrovert and gregarious. Furthermore, while the majority of calendrical savants are incapable of explaining exactly what strategies they follow (e.g., Heavey et al. (2012)), YV can explain in an articulate manner his strategy for calculating weekdays, and has detailed it in a separate publication [30]. His approach is based on structural knowledge of the Gregorian calendar and a dedicated algorithm involving an average of four additions and three divisions per problem.

The study was approved by the BSMS Research, Governance & Ethics Committee. The participant was informed about the study procedures and gave written consent to take part in the experiment.

### Experimental Tasks

#### Calendrical task

**Figure 1:**
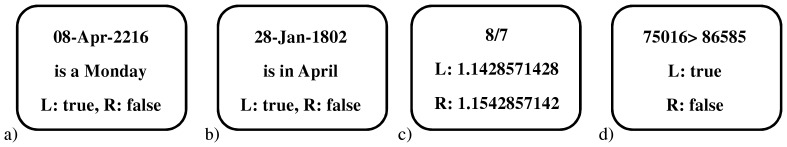
Example trials for the calendrical task (a) and its control (b), and for the division task (c) and its control (d) as they appeared on the screen. L and R, correspond to left and right response button respectively. See section 2.2 Methods, Experimental tasks, for detailed descriptions.

We adopted the tasks developed by [5]. The calendrical task featured dates from three periods of varying remoteness: 1) close dates, ranging between 1970–1989 and 2010–2029, 2) intermediate dates, ranging between 1900–1910 and 2100–2110, and 3) remote dates, ranging between 1753–1800 and 2200–2269. Dates were presented in text format, e.g. “07-Feb-1972 is a Monday; L: true, R: false?” (Fig. 1a), through a back-projection screen and responses were collected by button-presses with the right hand (L: left response button, R: right response button). The control condition for this task consisted of confirming whether a given date fell in a certain month, e.g. “28-Jan-1802 is in April; L: true, R: false?” (Fig. 1b). All task items concerned Monday, and correct statements were presented with a probability of 50%. The task was practiced outside the scanner prior to imaging. After scanning, the participant rated all presented dates on a difficulty scale with 0: easy, 1: challenging and 2: very difficult; this step was performed off-line to avoid confounding brain activity with processes related to the generation of a subjective rating.

#### Division task

To evaluate the anatomical substrates for division, taken as a representative complex mathematical operation, an additional task was devised, where integers between 1–9 were presented as operands, and YV was prompted to indicate the correct ratio between two alternatives, displayed with 10 decimal places, e.g. “8/7, L: 1.1428571428 R: 1.1542857142” (Fig. 1c). The control condition for this task consisted of direct comparison of two integers, which were longer to limit differences in reaction time e.g. “75016>86585, L: true, R: false” (Fig. 1d).

### Data acquisition

Imaging was performed on a 1.5 Tesla MR scanner (Magnetom Avanto, Siemens AG, Erlangen, DE) equipped with a standard head matrix coil at the Clinical Imaging Sciences Centre (CISC), University of Sussex. Functional images sensitized to blood-oxygen level-dependent (BOLD) contrast were obtained using T2*-weighted echo-planar sequences with TR = 2,620 ms, TE = 43 ms, 3×3 mm in-plane resolution, 64×64 matrix, 34 slices, 3 mm thickness, 20% gap; slice tilt was approximately 30 degrees to the bi-commissural plane and posterior-to-anterior phase encoding was chosen, to minimize susceptibility artefacts in frontal and temporal regions. Volumetric anatomical images were also obtained, using sagittal magnetization-prepared rapid acquisition gradient echo sequence with TR = 1,160 ms, TE = 4 ms, TI = 600 ms, FoV 230×230, matrix size 256×256, 192 slices with 0.9 mm thickness. Visual inspection of these scans on separate planes and 3D rendering by a senior neuroradiologist did not reveal any atypical anatomical variation in cortical gyration.

Both tasks were delivered in a block design, where the duration of each block was fixed to 24 s; within each block, stimulus presentation was self-paced, with a new stimulus appearing immediately after each response. For the calendrical task, 8 blocks were delivered for each date type and for the control condition, and a total of 370 functional volumes were acquired. For the division task, 8 blocks were delivered for the active and control conditions, and a total of 270 volumes were acquired.

In order to monitor autonomic arousal, taken as a proxy of effort [31], the continuous heart rate was monitored through an MRI-compatible pulse oximeter (Nonin 5400, Nonin Inc., Plymouth MN, USA).

### Data analysis

Using the routines provided in SPM8 (Wellcome Centre for Neuroimaging, London, UK), functional scans were slice-timing corrected, realigned and unwarped, and co-registered with the anatomical volume; normalization to Montreal neurological institute (MNI) space was then attained by iterative segmentation and realignment to template.

For univariate voxel-based analyses, functional data were smoothed with a Gaussian kernel having 8 mm full width at half-maximum. The experimental boxcars were convolved with the haemodynamic response function to obtain regressors for the active conditions (3 for the calendrical task, 2 for the division task - one division condition was discarded for technical reasons); the resting condition was implicitly modelled, and the 6 movement parameters were added as nuisance covariates. For succinctness, only imaging data from the close and remote dates is reported here, as intermediate dates reflected a “mixture” of the strategies adopted for those two (see below).

For the connectivity analyses, un-smoothed data were utilized as described elsewhere [22], [21] to avoid inducing artificial co-variance between adjacent regions. Based on existing literature, the following regions-of-interest (ROIs) were considered as potentially involved in the tasks of interest: anterior, middle and posterior cingulate, orbitofrontal, dorsomedial-, ventromedial-, anterolateral-, dorsolateral-, ventrolateral- prefrontal cortices, rostral and caudal supplementary motor area, anterior insula, inferior and superior parietal lobule, angular gyrus, supramarginal gyrus, precuneus, cuneus, temporal pole, anterior, middle, posterior inferior temporal cortex, anterior, middle, posterior middle temporal cortex, parahippocampal gyrus, hippocampus, anterior and posterior superior temporal sulcus, lingual gyrus, anterior, middle, posterior fusiform gyrus. The corresponding ROIs were derived from further subdivisions of those defined in the automated anatomical labelling atlas [32] (full list of abbreviations, co-ordinates and volumes provided in Table 1). To reduce partial volume effects, the ROIs were intersected with the individual brain mask; de-trending with a 3rd-degree polynomial was performed, followed by removal of covariance with movement vectors, global and cerebrospinal fluid signal.

**Table 1:** Regions of Interest (ROIs).

*Name*	*Description*	*Centroid co-ordinates (mm)*	*Vol. (ml)*
rACC	Anterior cingulate cortex, rostral part	(±6.4, 42.4, 4.5)	6.5
cACC	Anterior cingulate cortex, caudal part	(±5.7, 28.1, 25.7)	4.6
MCC	Middle cingulate cortex	(±7.0, −16.5, 40.3)	13.1
pCC	Posterior cingulate cortex	(±6.2, −44.2, 22.5)	3.1
OFC	Orbitofrontal cortex	(±15.5, 40.8, −17.5)	15.7
dmPFC	Dorsomedial prefrontal cortex	(±6.1, 3 8.0, 43.0)	9.3
vmPFC	Ventromedialprefrontal cortex	(±7.6, 58.3, 14.6)	9.2
alPFC	Anterolateral prefrontal cortex	(±27.6, 51.6, 18.3)	26.6
dlPFC	Dorsolateral prefrontal cortex	(±27.8, 18.0, 49.8)	32.6
vlPFC	Ventrolateral prefrontal cortex	(±46.7, 23.7, 14.1)	28.5
rSMA	Supplementary motor area, rostral part	(±6.0, 13.7, 59.1)	6.0
cSMA	Supplementary motor area, caudal part	(±7.0, −5.9, 63.2)	6.8
aINS	Anterior insula	(±34.0, 16.9, 0.9)	5.9
PARI	Inferior parietal lobule	(±43.1, −45.6, 47.1)	19.4
PARS	Superior parietal lobule	(±23.7, −59.5, 59.2)	16.5
ANG	Angular gyrus	(±43.7, −61.7, 37.7)	6.8
SMG	Supramarginal gyrus	(±55.8, −33.6, 30.5)	9.1
PREC	Precuneus	(±7.5, −56.9, 48.1)	22.5
CUN	Cuneus	(±8.5, −76.8, 28.5)	9.1
TPOLE	Temporal pole	(±39.8, 14.2, −26.0)	12.1
rITG	Inferior temporal gyrus, rostral part	(±46.2, −3.5, −35.2)	7.7
mITG	Inferior temporal gyrus, middle part	(±51.6, −30.1, −22.4)	9.7
cITG	Inferior temporal gyrus, caudal part	(±54.2, −51.9, −13.7)	8.6
rMTG	Middle temporal gyrus, rostral part	(±56.0, −7.5, −20.1)	10.0
mMTG	Middle temporal gyrus, middle part	(±57.5, −32.4, −4.4)	13.4
cMTG	Middle temporal gyrus, caudal part	(±53.4, −56.0, 7.3)	14.2
PHPP	Parahippocampal gyrus	(±24.8, −19.4, −24.5)	10.1
HPP	Hippocampus	(±29.3, −22.7, −14.8)	8.7
rSTG	Superior temporal gyrus, rostral part	(±51.1, −8.6, −2.3)	11.5
cSTG	Superior temporal gyrus, caudal part	(±53.6, −34.5, 12.9)	9.5
LING	Lingual gyrus	(±15.5, −67.6, −4.6)	16.8
rFG	Fusiform gyrus, rostral part	(±28.2, −5.1, −38.0)	4.1
mFG	Fusiform gyrus, middle part	(±30.9, −40.7, −18.1)	6.1
cFG	Fusiform gyrus, caudal part	(±34.2, −62.1, −14.1)	7.4

ROIs derived from the Automated Anatomical Labelling atlas [32] used for time-course extraction. Centroid co-ordinates, expressed in millimetres, refer to MNI space. All ROIs are symmetric between hemispheres. Volumes are given in millilitres.

We adopted an unbiased “network discovery” approach implemented in the form of pair-wise psychophysiological interaction (PPI) analysis [33] among all possible combinations of regions. This approach overcomes the restrictions inherent in choice of single seed regions in traditional PPI, and thereby provides full insight into the topological architecture of the effective connectivity network with minimal prior assumptions. To this end, separate sequential multi-linear regressions were performed for each pair of putative “source” and “target” regions, according to
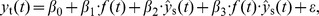
where 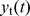 represents BOLD signal in the target region, 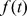 the regressor modelling the experimental condition, and 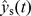 BOLD signal in the source region from which the linear effect of 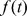 has been removed. Here, 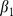 models the direct haemodynamic engagement of target region, 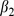 its intrinsic connectivity with the source region and 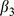 the effective connectivity, i.e. the modulation of connectivity conditional to the experimental condition, which was expanded with both f(t)∈[0,1] and f(t)∈[−1,1]. Non-linearity of the effective connectivity term results in a non-commutative group, making it possible to establish directional inferences on effective connectivity between regions. The potential of this modelling approach has been previously demonstrated in the context of block-design as well as event-related tasks [21], [22].

In order to obtain easily interpretable connectivity graphs, we performed the following contrasts: 1) Close dates vs. Date control task, 2) Remote dates vs. Date control task and 3) Division vs. Division control task. These analyses enabled us to explicitly map the most interconnected regions, i.e. the cortical hubs, supporting reasoning under the three conditions.

Following determination of the adjacency matrices for effective connectivity, the corresponding graphs were visualized using the Gephi 0.8 program (The Gephi Consortium, Paris, FR). In order to formally test whether certain nodes had a number of afferent connections (i.e., indegree) higher than would be predicted for a random network, 30 Erdös–Rényi random graphs were generated on the same nodes defined by the parcellated anatomical regions and matched for completeness. The indegree of each region was thereafter compared with that of the random graphs through one-sample t-tests Bonferroni-corrected accounting for multiple comparisons over regions.

## Results

### Behavioural and physiological responses

**Figure 2:**
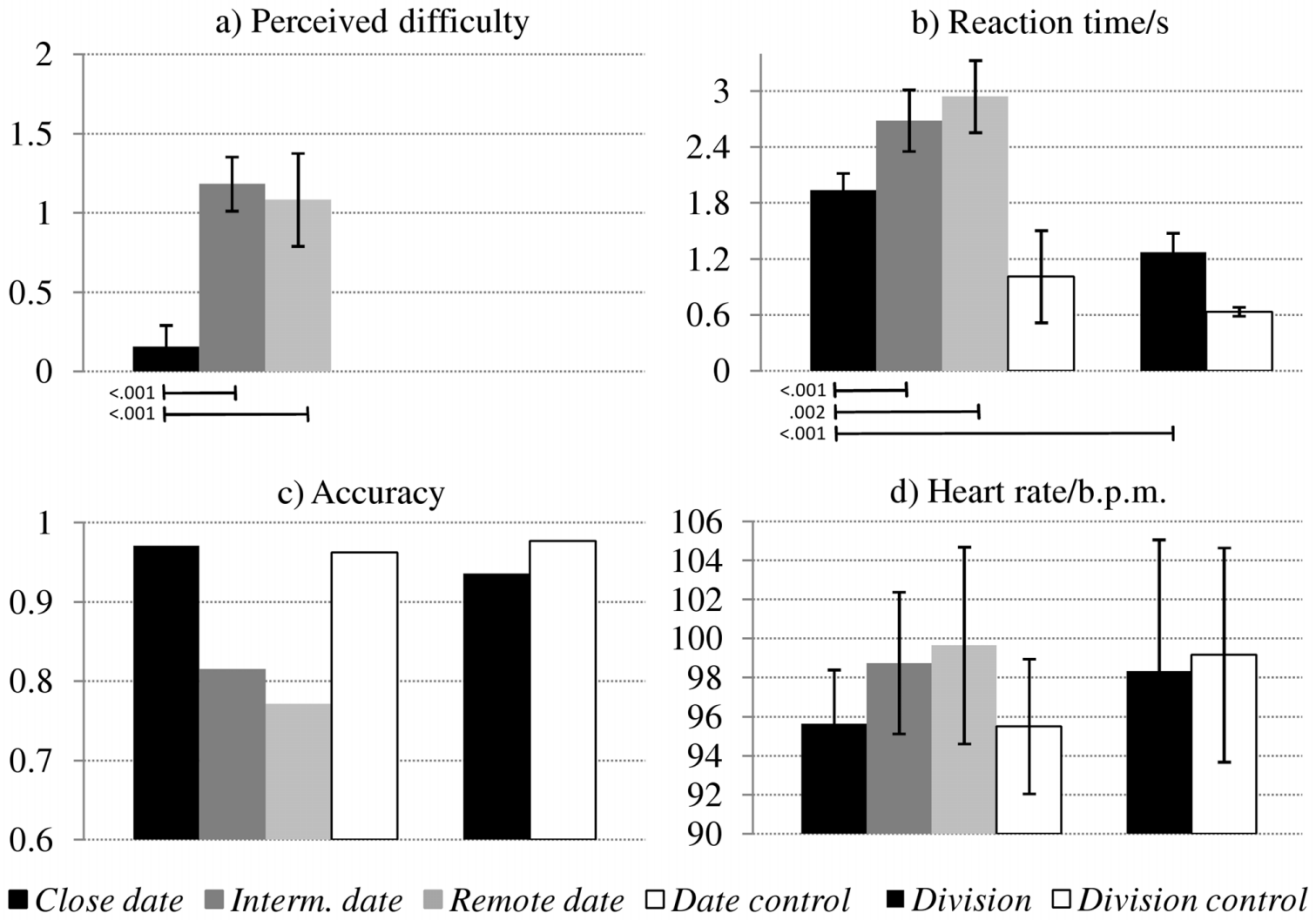
Behavioural and physiological responses for the different tasks and difficulty levels. (**a**) Perceived difficulty of calendric trials based on YV's ratings after the scan (scale 0–2, where 0 is easy, 1: challenging, 2: very difficult). (**b**) Reaction times in seconds. First 3 bars correspond to calendric task, 4^th^ bar to the calendric control task, 5^th^ to division task and 6^th^ to division control task. (**c**) Response accuracy for close dates: 97%, for intermediate dates: 82%, for remote dates: 77%, for the control task: 96%. Response accuracy for the division task was comparable to the performance for close dates at 94%, while accuracy for the control division condition was at 98%. (**d**) Heart rate in beats per minute (b.p.m.). Blocks containing close dates were perceived as significantly less challenging that those presenting intermediate or remote dates, and elicited shorter reaction times. Heart rate closely followed this pattern, but differences did not reach statistical significance. Error bars denote 1 standard deviation across blocks. Significance values are Bonferroni corrected over condition comparisons.

There were significant differences in the perceived difficulty of the date ranges: intermediate and remote dates were rated as significantly more difficult than close dates (unpaired t-test p<0.001 for both), but there was no difference between intermediate and remote dates (Fig. 2a). We observed a graded reaction time effect with significantly longer responses for remote and intermediate than close dates; even for the latter, the reaction time was longer than for divisions (Fig. 2b). Accordingly, the participant completed 103 close date, 76 intermediate date, 70 remote date and 156 division trials. Response accuracy was highest for close dates (97%), lower for intermediate dates (82%) and lowest for remote dates (77%); notably, the accuracy in the control task (96%) did not reach ceiling level, possibly due to response mapping errors. For the division task, the accuracy was similar to close dates (94%), and that of the corresponding control task similar to the calendrical control task (98%; Fig. 2c). Even though the differences did not reach statistical significance, the heart rate increased gradually with date distance (Fig. 2d).

### Univariate analyses

**Figure 3:**
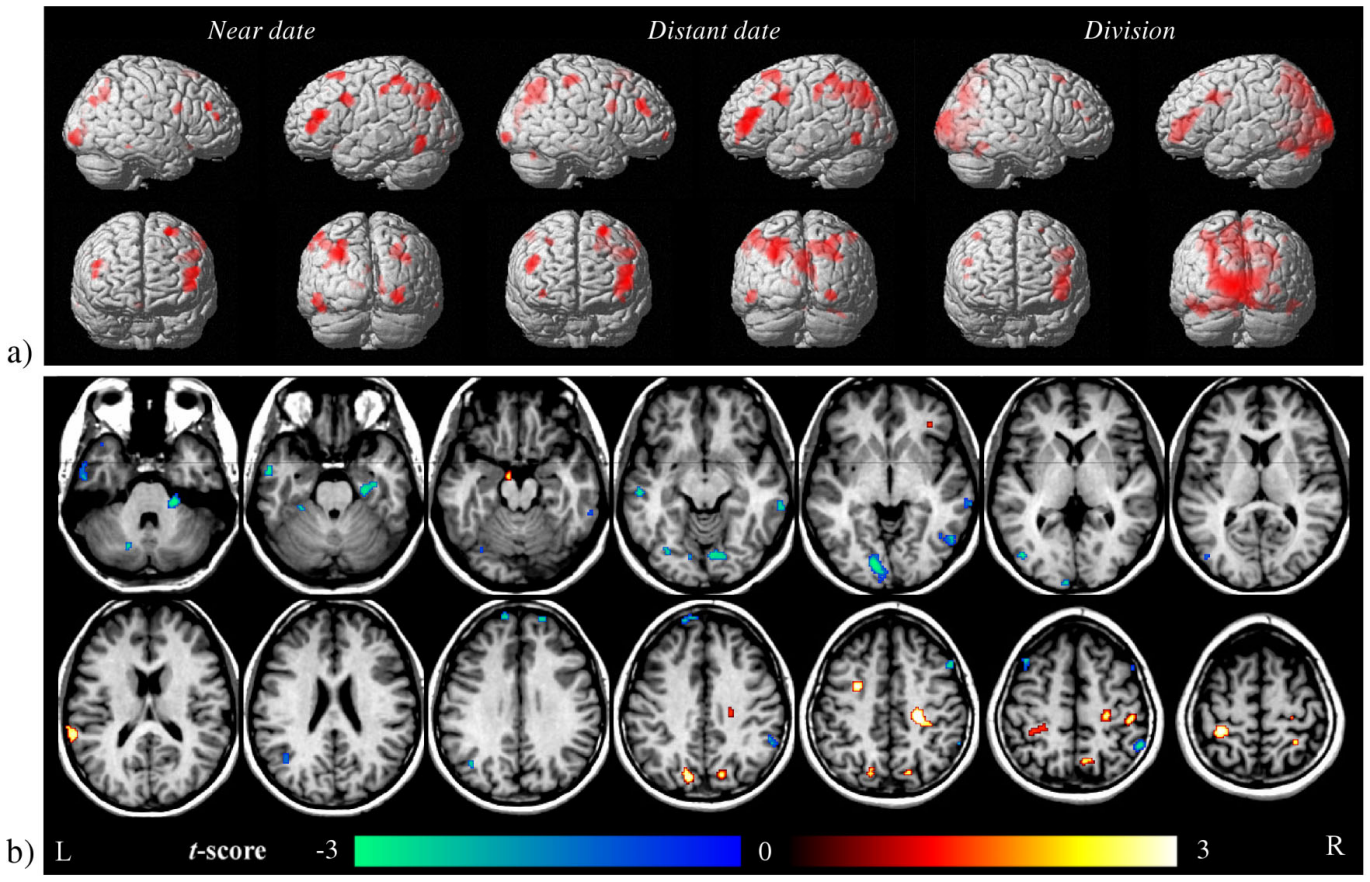
Univariate analysis results. a) Comparison of close, remote dates (calendrical task) and division task with their respective control conditions, shown at p<.001 un-corrected. See also Table 2. b) Comparison of close and remote date calculation trials, shown at p<0.01 uncorrected. Cold colours represent the contrast close>remote, warm colours the contrast remote>close. The sections shown correspond to MNI z = −33, −26 … 58 mm. R: right hemisphere, L: left hemisphere. See also Table 3.

The main effects of close date, remote date and division conditions are shown in Fig. 3a and corresponding clusters are given in Table 2. For all three conditions, the activation pattern appeared predominantly left-lateralized. Activations elicited by close and remote dates were largely overlapping, and located in the inferior and superior parietal lobules, angular gyrus, precuneus, inferior and middle frontal gyri, fusiform gyrus and occipital gyri. Similarly, for the division task activation clusters were primarily found in the cuneus, middle and inferior frontal gyri, cingulate and fusiform gyrus.

**Table 2:** Comparison of activity for calendrical tasks and division with their respective control tasks.

Peak (mm)	Vol. (ml)	Peak Z	p	Side	BA	Description
**Close date>Date Control**						
(−32, −74, 40)	7.46	5.4	<.001	L	BA40	inferior parietal lobule
(−24, −64, 32)		4.9	<.001	L	BA7	precuneus
(−56, −28, 46)		4.9	<.001	L	BA2	postcentral gyrus
(−46, 38, 18)	3.73	5.4	<.001	L	BA46	middle frontal gyrus
(−46, 44, 6)		5.1	<.001	L	BA46	middle frontal gyrus
(−48, 8, 36)	2.12	5.1	<.001	L	BA9	dorsolateral prefrontal cortex
(−34, 4, 32)		4	<.001	L	BA9	dorsolateral prefrontal cortex
(26, −66, 44)	2.45	4.8	<.001	R	BA7	superior parietal lobule
(38, −78, 36)		3.9	<.001	R	BA19	middle occipital gyrus
(32, −62, 54)		3.4	<.001	R	BA7	superior parietal lobule
(−50, −64, −8)	1.62	4.6	<.001	L	BA19	inferior temporal gyrus
(−46, −62, −18)		4	<.001	L	BA37	fusiform gyrus
(−26, 16, 62)	1.13	4.4	<.001	L	BA6	middle frontal gyrus
(−22, 26, 60)		4	<.001	L	BA6	superior frontal gyrus
(26, −96, −8)	1.57	4.4	<.001	R	BA18	lingual gyrus
(32, −92, 2)		3.6	<.001	R	BA18	middle occipital gyrus
(14,−86,0)	0.49	4.3	<.001	R	BA17	calcarine gyrus
(12,−88,12)		3.4	<.001	R	BA17	calcarine gyrus
(48, 8, 24)	0.93	4.2	<.001	R	BA44	inferior frontal gyrus
(36, 8, 24)		3.4	<.001	R	BA13	insula
(46,40,28)	0.38	4.2	<.001	R	BA46	middle frontal gyrus
(6,20,−16)	0.25	3.7	<.001	R	BA25	subcallosal cingulate gyrus
(−16,−58,6)	0.29	3.7	<.001	L	BA17	lingual gyrus
**Remote date>Date Control**						
(−26, −64, 32)	27.36	7	<.001	L	BA39	angular gyrus
(26, −66, 44)		6.5	<.001	R	BA7	superior parietal lobule
(−56, -28, 46)		5.8	<.001	L	BA2	postcentral gyrus
(−48, 44, 6)	6.72	7	<.001	L	BA46	Inferior frontal gyrus
(−46, 38, 18)		6.5	<.001	L	BA46	middle frontal gyrus
(−44, 54, −12)		3.3	<.001	L	BA11	middle frontal gyrus
(−48, 8, 36)	7.28	5.9	<.001	L	BA9	dorsolateral PFC
(−24, 16, 60)		5.1	<.001	L	BA6	middle frontal gyrus
(−22, 24, 60)		4.8	<.001	L	BA6	superior fontal gyrus
(44, 38, 28)	1.7	5.5	<.001	R	BA46	middle frontal gyrus
(48, 44, 20)		4	<.001	R	BA46	middle frontal gyrus
(−50, −66, −6)	1.03	5.3	<.001	L	BA19	inferior temporal gyrus
(48, 8, 24)	1.12	5	<.001	R	BA44	inferior frontal gyrus
(48, −36, 54)	1.38	4.7	<.001	R	BA40	postcentral gyrus
(26, −98, −8)	0.76	4.7	<.001	R	BA18	lingual gyrus
(−8,−86,6)	0.42	4.2	<.001	L	BA17	cuneus
(26,8,48)	0.57	3.8	<.001	R	BA6	middle frontal gyrus
(4,−34,40)	0.58	3.8	<.001	R	BA31	cingulate gyrus
(−4,−32,26)		3.5	<.001	L	BA23	posterior cingulate
(34,62,−6)	0.24	3.7	<.001	R	BA10	superior orbital gyrus
(38,−70,−28)	0.39	3.5	<.001	R		cerebellum (Lobule VIIa)
(26,−70,−22)		3.3	<.001	R		Cerebellum (Lobule VI)
**Division date>Division Control**						
(−6, −96, 4)	90.86	15.7	<.001	L	BA18	cuneus
(−16, −100, 6)		13.5	<.001	L	BA18	cuneus
(−12, −84, −4)		12.8	<.001	L	BA18	lingual gyrus
(−44, 42, 6)	7.22	6.8	<.001	L	BA46	middle frontal gyrus
(−44, 54, −8)		5.1	<.001	L	BA11	middle frontal gyrus
(−46, 32, 18)		4.9	<.001	L	BA46	middle frontal gyrus
(−46, 10, 36)	4.51	5.3	<.001	L	BA9	dorsolateral PFC
(−40, 16, 36)		5	<.001	L	BA9	middle frontal gyrus
(−34, 0, 40)		4	<.001	L	BA6	middle frontal gyrus
(46, −64, −22)	1.32	4.7	<.001	R	BA37	fusiform gyrus
(50, −58, −16)		4.4	<.001	R	BA37	fusiform gyrus
(40,16,58)	0.66	4.5	<.001	R	BA6	middle frontal gyrus
(24,−28,−8)	0.62	4.2	<.001	R	BA27	parahippocampal gyrus
(34,−26,−6)		3.5	<.001	R	BA13	insula
(48,38,26)	0.58	3.8	<.001	R	BA46	middle frontal gyrus
(−4, −42, 16)	0.75	3.8	<.001	L	BA30	posterior cingulate
(0, −38, 22)		3.7	<.001	R	BA31	cingulate gyrus

A voxel-level threshold of p<.001 uncorrected was applied (min. 30 voxels cluster extent). Peak co-ordinates, expressed in millimetres, refer to MNI space.

Even at the permissive voxel-level threshold of p<0.01 uncorrected, direct comparison of close and remote dates only revealed limited and scattered activation differences (Fig. 3b and Table 3). Close dates elicited larger BOLD responses mainly in early visual areas (significant at cluster level), cuneus, right parahippocampal gyrus and left medial temporal lobe; the converse was true for cingulate (significant at cluster level), postcentral gyrus, precuneus, middle and inferior frontal gyri.

**Table 3:** Comparison of activity for remote and close dates.

Peak (mm)	Vol. (ml)	Peak Z	p	Side	BA	Description
**Remote date>Close date**						
(24, −26, 44)	1.45*	3.5	p<.001	R	BA31	Cingulate gyrus
(36, −32, 44)		2.5	p = .007	R	BA2	Postcentral gyrus
(−34, −38, 56)	0.95	3.2	p<.001	L	BA40	Postcentral gyrus
(−28, 2, 42)	0.36	3.2	p<.001	L	BA6	Middle frontal gyrus
(−14, −76, 40)	0.68	3.1	p<.001	L	BA7	Precuneus
(16, −76, 40)	0.42	3	p = .001	R	BA7	Precuneus
(−64, −40, 14)	0.49	3	p = .001	L	BA45	Inferior frontal gyrus
(46, −28, 54)	0.42	2.9	p = .002	R	BA2	Postcentral gyrus
(34, 34, −4)	0.08	2.8	p = .003	R	BA47	Inferior frontal gyrus
(14, −62, 52)	0.22	2.7	p = .003	R	BA7	Precuneus
(−10, −10, −18)	0.12	2.6	p = .005	L	BA34	Parahippocampal gyrus
(32, −48, 56)	0.11	2.5	p = .006	R	BA7	Precuneus
**Close date>Remote date**						
(−14, −84, −6)	1.31*	3.2	p<.001	L	BA18	Lingual gyrus
(−6, −92, −8)		2.9	p = .002	L	BA17	Lingual gyrus
(−8, −102, 0)		2.6	p = .005	L	BA17	Cuneus
(22, −32, −32)	0.94	3	p<.001	R	BA35	Parahippocampal gyrus
(26, −22, −26)		2.8	p = .003	R	BA35	Parahippocampal gyrus
(34, −18, −26)		2.5	p = .006	R	BA36	Parahippocampal gyrus
(−56, −2, −28)	0.57	3	p = .001	L	BA21	Middle temporal gyrus
(−44, −78, 4)	0.27	3	p = .001	L	BA19	Middle occipital gyrus
(52, 20, 48)	0.25	2.9	p = .002	R	BA8	Middle frontal gyrus
(−18, −70, −34)	0.11	2.8	p = .002	L		Cerebellum (Lobule VIIa Crus I)
(−40, −64, 26)	0.33	2.8	p = .002	L	BA39	Middle temporal gyrus
(54, −50, 52)	0.27	2.8	p = .002	R	BA40	Inferior parietal lobule
(−38, 18, −36)	0.1	2.8	p = .003	L	BA38	Superior temporal gyrus
(−56, −24, −12)	0.18	2.8	p = .003	L	BA21	Middle temporal gyrus
(−28, −36, −24)	0.11	2.7	p = .003	L	BA20	Fusiform gyrus
(−12, 60, 32)	0.41	2.7	p = .003	L	BA10	Frontopolar prefrontal cortex
(52, −64, −2)	0.4	2.7	p = .003	R	BA37	Middle temporal gyrus
(44, −62, −4)		2.5	p = .005	R	BA37	Middle temporal gyrus
(66, −34, −8)	0.51	2.7	p = .004	R	BA21	Middle temporal gyrus
(60, −42, −18)		2.3	p = .01	R	BA20	Inferior temporal gyrus
(6, −78, −12)	0.31	2.7	p = .004	R	BA18	Lingual gyrus
(−56, −48, −10)	0.24	2.7	p = .004	L	BA37	Middle temporal gyrus
(−32, −74, −14)	0.22	2.7	p = .004	L	BA19/37	Fusiform gyrus
(−40, 24, 52)	0.19	2.6	p = .004	L	BA8	Middle frontal gyrus
(−42, 16, 52)		2.4	p = .007	L	BA8	Middle frontal gyrus
(20, 60, 32)	0.14	2.6	p = .004	R	BA10	Frontopolar prefrontal cortex
(58, −46, 40)	0.23	2.6	p = .005	R	BA40	Inferior parietal lobule

A voxel-level threshold of p<.01 un-corrected was applied. Superscript “*” next to cluster volumes (expressed in millilitres) denotes cluster-level significance at p<.01 un-corrected (min. 10 voxels cluster extent). Peak co-ordinates, expressed in millimetres, refer to MNI space.

### Effective connectivity

**Figure 4:**
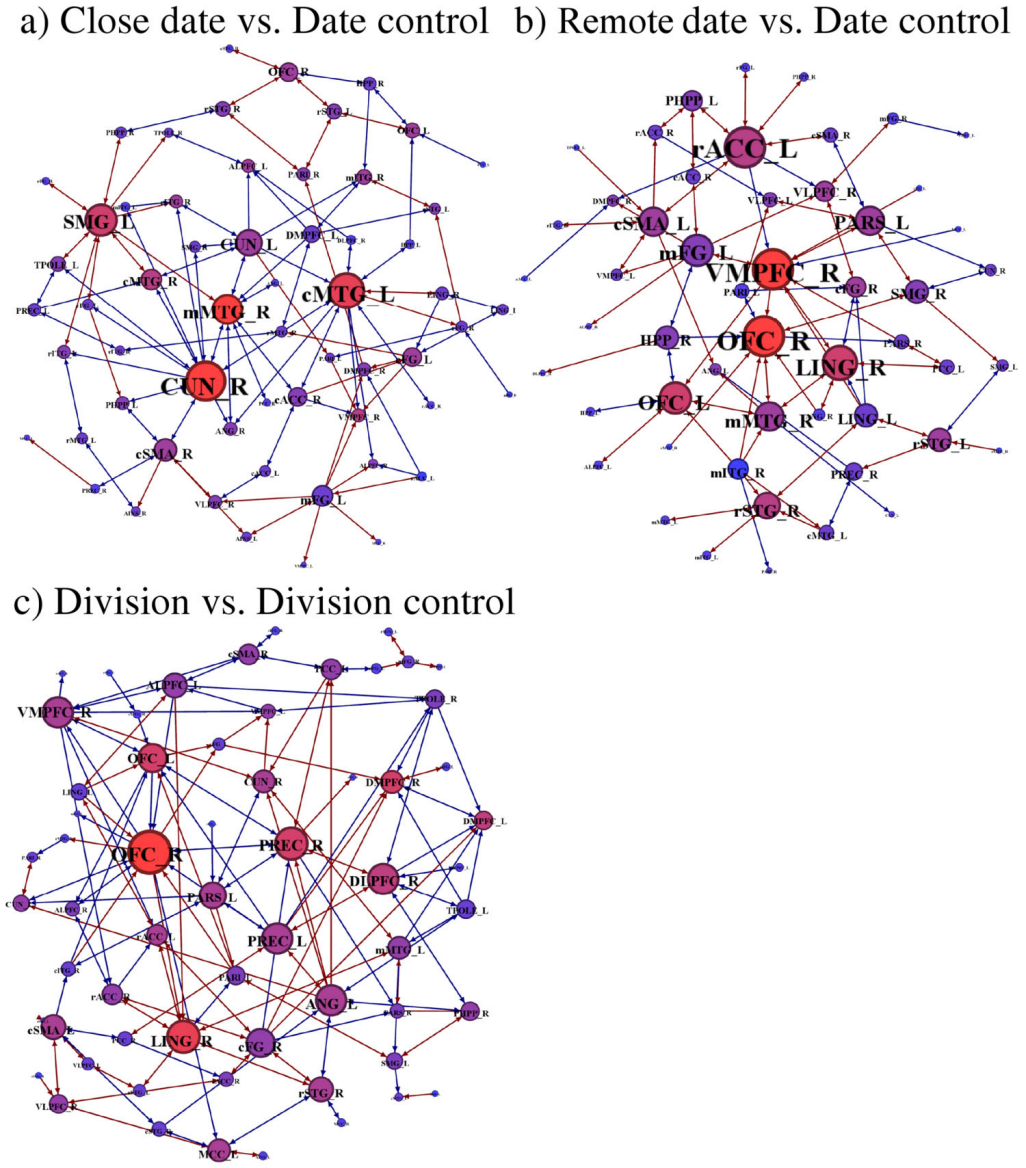
Effective connectivity for close, remote dates and division with the respective control conditions (a, b and c). Node diameter represents degree (total number of connections), whereas node colour (blue through red) encodes indegree (number of significant entrant connections). CUN: cuneus, MTG: middle temporal gyrus, SMG: supramarginal gyrus, OFC: orbitofrontal cortex, VMPFC: ventromedial prefrontal cortex, rACC: anterior cingulate cortex, LING: lingual gyrus, PARS: superior parietal lobule, PREC: precuneus. See Table 1 for full list of abbreviations.

Effective connectivity networks for close date, remote date and division conditions are shown in Fig. 4a, b and c respectively; corresponding regions displaying an indegree higher than random graphs are given in Table 4. For close dates, the highest indegree was observed for the right cuneus (CUN_R),right central third and left posterior part of middle temporal gyrus (mMTG_R and cMTG_L), left supramarginal gyrus (SMG_L) and right ventromedial prefrontal cortex (Fig. 4a). For remote dates, the indegree was highest for the right and left orbitofrontal cortex (OFC_L and OFC_R), right ventromedial prefrontal cortex (VMPFC_R), right lingual gyrus and left anterior cingulate cortex (rACC_L) (Fig. 4b).

**Table 4:** Brain regions with indegree higher than random graphs.

ROI name	Observed in-degree	E-R in-degree	p
**Close date>Date control**			
CUN_R	8	2.1±1.0	p<.001, t(29) = 32.5
mMTG_R	8	2.5±1.6	p<.001, t(29) = 19.1
cMTG_L	7	2.6±2.1	p<.001, t(29) = 11.6
SMG_L	6	2.2±1.4	p<.001, t(29) = 15.1
VMPFC_R	5	2.0±1.2	p<.001, t(29) = 13.3
cMTG_R	5	2.3±1.4	p<.001, t(29) = 10.6
OFC_L	4	1.8±1.3	p<.001, t(29) = 8.8
ALPFC_L	4	2.1±1.2	p<.001, t(29) = 8.6
cFG_L	4	2.3±1.1	p<.001, t(29) = 8.3
OFC_R	4	2.1±1.3	p<.001, t(29) = 8.0
CUN_L	4	2.2±1.4	p<.001, t(29) = 7.3
mITG_R	4	2.1±1.5	p<.001, t(29) = 7.1
cITG_R	4	2.0±1.6	p<.001, t(29) = 6.8
cSMA_R	4	2.7±1.6	p = .009, t(29) = 4.4
rSTG_R	3	1.9±1.2	p<.001, t(29) = 4.8
ALPFC_R	3	1.9±1.3	p = .003, t(29) = 4.7
cACC_R	3	2.0±1.2	p<.001, t(29) = 4.4
Completeness	0.034	0.032±0.002	p = 0.2
**Remote date>Date control**			
VMPFC_R	8	1.7±1.4	p<.001, t(29) = 25.2
OFC_R	8	1.8±1.4	p<.001, t(29) = 24.7
OFC_L	6	1.3±1.4	p<.001, t(29) = 17.9
LING_R	6	2.0±1.4	p<.001, t(29) = 15.5
rACC_L	5	2.0±1.3	p<.001, t(29) = 12.8
rSTG_R	5	2.0±1.5	p<.001, t(29) = 10.7
VLPFC_R	4	1.6±1.2	p<.001, t(29) = 11.4
cFG_R	4	1.6±1.3	p<.001, t(29) = 10.7
mMTG_R	4	1.6±1.3	p<.001, t(29) = 10.3
rSTG_L	4	1.6±1.3	p<.001, t(29) = 10.2
PARS_L	4	1.7±1.3	p<.001, t(29) = 9.9
cSMA_L	4	1.8±1.4	p<.001, t(29) = 8.9
DMPFC_R	3	1.6±1.0	p<.001, t(29) = 7.8
HPP_R	3	1.7±1.0	p<.001, t(29) = 7.5
PHPP_L	3	1.6±1.2	p<.001, t(29) = 6.4
ANG_L	3	1.7±1.3	p<.001, t(29) = 5.5
SMG_R	3	1.6±1.4	p<.001, t(29) = 5.5
VLPFC_L	3	1.6±1.4	p<.001, t(29) = 5.4
mFG_L	3	1.8±1.2	p<.001, t(29) = 5.3
Completeness	0.026	0.025±0.003	p = 0.3
**Division>Division control**			
OFC_R	9	2.9±1.8	p<.001, t(29) = 19.1
LING_R	8	2.6±1.4	p<.001, t(29) = 20.3
DMPFC_R	7	2.5±1.5	p<.001, t(29) = 15.9
PREC_R	7	2.9±1.8	p<.001, t(29) = 12.4
OFC_L	7	3.0±1.8	p<.001, t(29) = 12.2
DLPFC_R	6	2.2±1.3	p<.001, t(29) = 16.4
DMPFC_L	6	2.7±1.2	p<.001, t(29) = 14.6
CUN_R	5	2.5±1.3	p<.001, t(29) = 10.1
PARS_L	5	2.6±1.5	p<.001, t(29) = 8.7
VMPFC_R	5	2.9±1.4	p<.001, t(29) = 8.4
PREC_L	5	2.6±1.5	p<.001, t(29) = 8.4
rSTG_R	5	3.0±1.4	p<.001, t(29) = 7.7
rACC_L	5	3.0±1.5	p<.001, t(29) = 7.6
cSMA_L	5	3.0±1.8	p<.001, t(29) = 6.0
ANG_L	5	2.9±2.3	p = .002, t(29) = 5.0
MCC_L	5	3.1±2.3	p = .008, t(29) = 4.4
cFG_R	4	2.3±1.5	p<.001, t(29) = 6.1
ALPFC_L	4	2.4±1.5	p<.001, t(29) = 5.7
VMPFC_L	4	2.7±1.4	p = .002, t(29) = 5.0
cSMA_R	4	2.5±1.7	p = .003, t(29) = 4.8
PCC_L	4	2.6±1.9	p = .03, t(29) = 4.0
PARI_L	3	2.3±1.0	p = .04, t(29) = 3.8
Completeness	0.041	0.04±0.003	p = 0.2

Comparison of indegree between the observed effective connectivity networks and 30 corresponding Erdös–Rényi (ER) random graphs matched for completeness. Only nodes having a number of incident connections significantly larger than the random graphs are listed. All p-values are Bonferroni-corrected accounting for 68 comparisons over the whole ROI set.

## Discussion

In this study, we tested whether the strategy adopted by a non-autistic calendrical expert would change as a function of level of practice with date range, and whether this would reflect in activation and connectivity patterns in his brain. Close and remote dates in the past and future differed substantially in level of practice (YV's own report), which reflected in perceived difficulty, response accuracy and reaction times. According to YV's own account, processing of close dates was facilitated by application of a number of “shortcuts” and reference to memorized associations, whereas the remote dates required additional steps including conversion to a suitable close date.

The univariate activation patterns for remote and close dates were largely overlapping and consistent with previous imaging studies of arithmetic processing(e.g., [34], [35]. Direct contrast of responses for remote and close dates revealed only small and scattered clusters even at a highly permissive threshold. We speculate that the greater engagement of parahippocampal and middle temporal gyri for close dates reflected encoding and retrieval of facts stored through extensive practice in LTM.

By contrast, network discovery based on pair-wise modelling of effective connectivity revealed a markedly different pattern for close and remote dates: we found a number of cortical hubs which displayed significant convergence of connections (even after Bonferroni correction) and were located in different brain regions. The computations for close dates largely depended on connectivity in posterior parietal and middle temporal areas. This finding likely reflects retrieval operations and tapping into the rich pre-existing associations between well-practiced dates and weekdays and stronger associations for practiced years [5]. Indeed, this result is in agreement with previous work on the effect of practice on activity of brain regions involved in representing Arabic numerals, and the mental number line [36], [37], as well as with the role of recognition memory and semantic memory in experts solving problems in the domain of their specialization (e.g., [38], see [8] for a review). These associations are used by expert mental calculators to achieve fast and accurate performance in three different ways: 1) meaningful encoding of patterns of numbers, which promotes their retention, 2) pattern recognition and selection of the most appropriate computation strategy for each particular problem, and 3) substituting computations with retrieval for intermediate results, where possible, decreasing solution times [10].

Connectivity analysis for remote dates revealed a network hinged around the orbitofrontal and ventral-medial prefrontal cortex. This enhanced prefrontal connectivity, also observed during the less practiced division trials, accords with reported modulation by task difficulty and arithmetic complexity, as well as with the established role of these areas in fast perceptual and cognitive processing of numbers [35]. Importantly, the remote dates involved an extra step of conversion to a corresponding practiced date, e.g. 26-Aug-2238 was converted to 26-Aug-1838 (YV's own report). This extra step increased calculation demands, and relied more on prefrontal control mechanisms for successful problem solving, possibly exerted on distributed representations of arithmetic operations in subdivisions of the parietal cortex [39]. This additional calculation reflected a different level of strategic reasoning on how the remote date trials were approached. It involved performance monitoring and resulted in a specific behavioural adjustment, which was not used in the close date trials. All these elements are consistent with selective engagement of the medial prefrontal and orbitofrontal cortex, as well as the rostral anterior cingulate, as reported in an elegant study that involved different levels of reasoning and strategic thinking [40]. Although that particular study entailed a task that required reasoning about others and decision making in an analogue of the “Beauty Contest” game in a competitive interactive setting, it still shared elements of adaptive learning and different levels of reasoning and complexity for different types of trials with our task. The emergence of the anterior cingulate as a major hub in the remote dates condition may also reflect increased connectivity in a component of a central executive system in the context of arithmetic calculations with increased working memory demands [41], [42]. Additionally, it may be related to increased regulation of autonomic function and effortful cognitive control, e.g. [43], in the context of increased task difficulty. Finally, high afferent connectivity in the lingual gyrus could reflect increased visual encoding and processing effort [44], as well as top-down feedback for enhanced processing of relevant stimulus features [45].

Our results extend the earlier PET results by Pesenti et al. (2001), which showed unique patterns of activation in a mental calculator compared to control participants, by revealing two engagement patterns in the same expert varying as a function of practice and familiarity of date range. The more practiced calculations engaged a network of cortical hubs, including right medial frontal and right medial temporal areas, consistent with providing support to long-term working memory in one's area of expertise [11], [9]. Earlier behavioural and theoretical work has suggested that extensive practice in a specific domain promotes the creation of knowledge structures and procedures for efficient encoding and retrieval of task-relevant information in LTM [10]. These acquired structures of information organised in familiar or regular forms, known as chunks, are stored in large numbers in LTM, and allow the expert to perform tasks with higher speed and accuracy than novices [46], [47], [9], [10]. These contributions have been recently corroborated with fMRI evidence; for instance, training aimed at increasing working memory capacity and acquisition of expert skills appears to depend on a common prefrontal-parietal network [48], [49], [50], as well as fusiform and parahippocampal areas that support recognition memory [38]. Notably, further work on encoding strategies involving chunking has demonstrated that activity in the lateral frontal cortex, which mediates both retrieval and integration of contextual associations [51], decreases in intensity with practice [52], [53].

Our study is limited by the fact that we have only tested one participant. Given, however, the scarcity of mental calculators, the heterogeneity of their overall cognitive abilities and of the strategies they use, we believe that the approach of a within-subject comparison is informative. It was particularly valuable that the participant was able to explain his calculation approach for the calendrical problems, and identify the different strategic reasoning involved in responding to remote dates. Due to limitations inherent in a single-subject approach and limited scanner field strength, the statistical significance of univariate analyses was inadequate to deliver conclusive results. Yet, effective connectivity analysis provided a convincing set of findings, which survived stringent correction for multiple comparisons, and corresponded to different neural contexts which were evoked by the practiced and non-practiced dates.

Similarly to the way in which the study of synaesthesia, a rare condition (prevalence of 1∶2000), where a stimulus feature elicits the experience of a different attribute (e.g., the letter N is blue) can inform our understanding of conscious awareness, memory, creativity and so on [54], the study of mental calculators can further our understanding of the brain mechanisms underpinning the development of expertise. The fact that YV does not fall within the autistic spectrum suggests that his skills are not supported by neurobiological peculiarities and could be acquired by other people. This view is supported by the dramatic performance improvement in digit-span tasks [47] and mental calculations [10] afforded by systematic practice. Development of expertise has also been studied extensively in the domain of chess expertise, see [55] for a review. In a study of particular relevance involving chess experts of a similar level, Bilalic and colleagues tested problem-solving strategies for different game openings these experts specialized in [56]. The effect of this sub-specialization was stronger than their general chess expertise, and their strategy depended on their knowledge and familiarity with the particular problems. Here we propose that in mathematical expertise the ability to use long-term working memory can be developed in a way akin to a computer extending its “capacity of RAM by using swap space on the hard drive to create a larger ‘virtual memory’” [57]. We further speculate that when dealing with less familiar materials, and possibly in the early stages of acquiring the relevant expertise, engagement of supervisory regions supports the networks normally involved in the more automated execution of calculations. Finally, although it is beyond the scope of this paper to discuss the debate on deliberate practice vs. innate ability, e.g., [58], [59], our study does not provide evidence for specific innate ability for mental calculations. As put by Charles Darwin to Francis Galton: “[…] I have always maintained that excepting fools, men did not differ much in intellect, only in zeal and hard work; I still think this an *eminently* important difference.” [60], p. 290, quoted by [61].
